# Hans Wolfgang Sachs (1912–2000): From Nazi *Volkstumskämpfer* and chief pathologist of the Reich Physician SS to chairholder in the Federal Republic of Germany. English version

**DOI:** 10.1007/s00292-019-00737-z

**Published:** 2020-11-10

**Authors:** Jens Westemeier, Sebastian Scheib, Hendrik Uhlendahl, Dominik Gross, Mathias Schmidt

**Affiliations:** Institute for History, Theory and Ethics of Medicine, University Hospital, RWTH Aachen University, Wendlingweg 2, 52074 Aachen, Germany

**Keywords:** Chemical warfare agents, Concentration camps, National Socialism, Pathology, World War II, Chemische Kampfstoffe, Konzentrationslager, Nationalsozialismus, Pathologie, Zweiter Weltkrieg

## Abstract

During the Second World War, the German Wehrmacht and the SS tested various chemical warfare agents on prisoners of concentration camps. The SS needed a pathologist to do this. Therefore, Reichsarzt SS Ernst-Robert Grawitz recruited the 32-year-old Hans Wolfgang Sachs. Despite his position as senior pathologist at the office of the Reichsarzt SS, Sachs was spared interrogation and prosecution after 1945, although the prosecution presented a document about chemical warfare and human experiments during the Nuremberg medical trial. In this, Sachs was named as a participant in so-called “N-Stoff” (chlorine trifluoride) experiments. Little is known about Sachs to this day. This article is intended to close this gap. Of particular interest are the motives and reasons why Sachs joined the party and the SS, as well as his career after 1945.

## Abstract (de)

Die deutsche Wehrmacht und die SS testeten während des Zweiten Weltkriegs verschiedene chemische Kampfstoffe an KZ-Häftlingen. Dafür benötigte die SS auch einen Pathologen. Der Mann, den Reichsarzt-SS Ernst-Robert Grawitz dafür zum Übertritt in die Waffen-SS überzeugen konnte, war der erst 32-jährige Hans Wolfgang Sachs. Nach 1945 blieben Sachs trotz seiner Stellung als „Leitender Pathologe beim Reichsarzt-SS“ eine Vernehmung und Anklage erspart. Dabei hatte bereits die Anklagevertretung während des Nürnberger Ärzteprozesses im Zusammenhang mit den Kampfstoffversuchen ein Dokument vorgelegt, das Sachs namentlich als Teilnehmer an sog. N‑Stoff-Versuchen (Chlortrifluorid) auswies. So konnte er im Nachkriegsdeutschland bald wieder in der Wissenschaft Fuß fassen und schließlich sogar den Lehrstuhl für Gerichtliche Medizin an der Universität Münster besetzen. Bis heute ist über Sachs kaum etwas bekannt. Diese Lücke soll der vorliegende Beitrag schließen. Von besonderem Interesse sind hierbei einerseits die Motive und Gründe, aus denen sich Sachs der Partei und der SS anschloss, und andererseits der Karriereverlauf nach 1945.

## Background

**Fig. 1:**
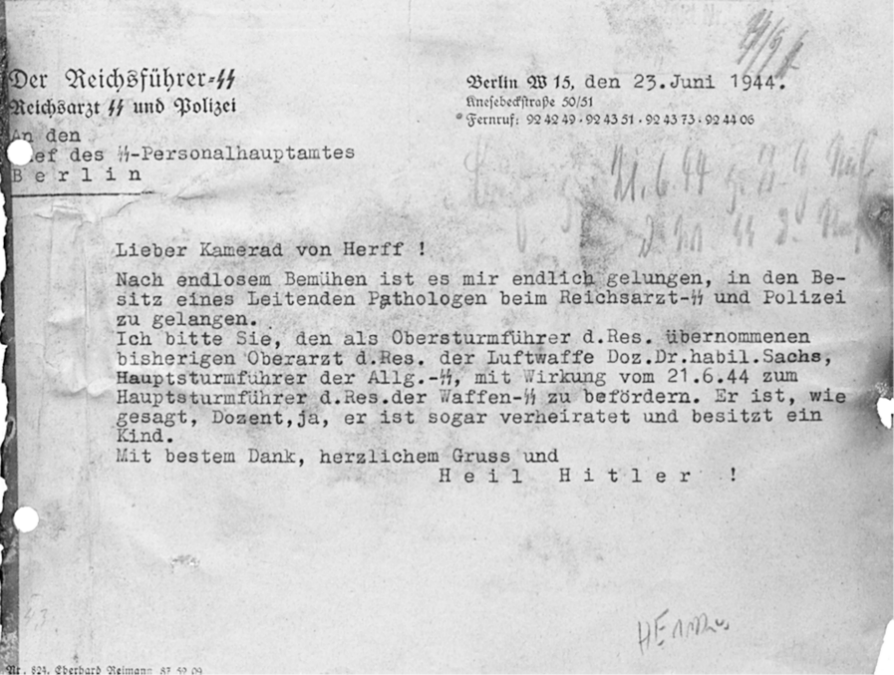
Grawitz’s letter of 23.06.1944 concerning the takeover of Sachs by the *Waffen-SS* [20]

In June 1944, after the successful landing of the Allies in Normandy and the destruction of the *Heeresgruppe Mitte* (central army group) by the Soviet army, the military defeat of the German Reich became clearly apparent. In this situation, *Reichsarzt SS* (Reich Physician SS) Ernst-Robert Grawitz (1899–1945), the chief physician within the SS, wrote to the head of the SS personnel main office that after “endless efforts,” he had finally succeeded in “getting into the possession of a chief pathologist at the Reichsarzt SS and Police.” SS member Hans Wolfgang Sachs, who worked as a physician in the Luftwaffe, was to be immediately transferred to the *Waffen-SS* (Fig. 1; [20]).

The man whom Grawitz was able to convince to join the Waffen-SS was the 32-year-old Hans Wolfgang Sachs. After 1945, despite his position as leading pathologist at the Reichsarzt SS, Sachs was not subjected to interrogation and accusation. This was despite the fact that the prosecution had already presented a document during the Nuremberg Medical Trial which mentioned Sachs by name and thus connected him to the so-called *N‑Stoff* experiments (tests with chemical warfare agents) [36]. Thus, he was soon able to regain a foothold in science in post-war Germany and eventually even filled the chair of forensic medicine at the University of Münster. To this day, hardly anything is known about Sachs. The present article is intended to fill this gap.

Of particular interest here are on the one hand, the motives and reasons for which Sachs joined the party and the SS, and on the other hand, his career path after 1945.

## Materials and methods

The work is based in central parts on original documents. The sources are the contemporary SS files from the Federal Archives Berlin (BArch) and documents from the Federal Archives Ludwigsburg (BAL) and the State Archives Ludwigsburg (StAL), as well as documents from the University (UAM) and City Archives of Münster, Westphalia. The primary sources mentioned above are supplemented by the research literature available to date on the history of warfare agent research and human experiments in the Third Reich and the history of the universities Münster and Prague.

## Childhood and youth

Hans Wolfgang Sachs was born on March 31, 1912, in Aussig (Czech: *Ústí nad Labem*) in the Sudetenland in Bohemia, at that time part of the Habsburg Monarchy [6, 11]. The industrial city on the Elbe, about 50 km south of Dresden, had more than 37,000 inhabitants at the beginning of the 20th century, most of whom were German speaking. Sachs came from a middle-class family. His father Johann was headmaster of the local grammar school and his mother Olga brought up their son according to the Protestant faith [6, 11]. With the beginning of World War I in 1914, tensions between Czechs and Sudeten Germans in Bohemia grew. After the disintegration of Austria-Hungary, the territory fell to the newly founded Czechoslovakia in October 1918. The German minority represented about one third of the population of the new country. They felt disadvantaged and as losers of the war. The ethnic conflict between the ethnic groups led to excessive nationalism and determined the political and social climate in which Sachs was socialized [34].

## Study and engagement as a political activist

In June 1930, Sachs passed his school-leaving examination at the *Staatsrealgymnasium* Aussig and subsequently began to study medicine at the German Charles University in Prague [6, 11] (the Prague university had been divided into two independent institutions, one German and one Czech, in 1882 [40]).

In the German Reich, Adolf Hitler and the National Socialist German Workers’ Party (NSDAP)—until then an insignificant splinter party—achieved 18.3% of the votes in the Reichstag elections on September 14, 1930, making them the second strongest force behind the Social Democratic Party of Germany (SPD). In Czechoslovakia in the 1920s, the originally “ethnic” German National Socialist Workers’ Party (DNSAP) developed into a fascist movement of the Sudeten Germans with similar aims to those of the NSDAP [39]. Sachs, by his own account, soon became involved in the National Socialist agenda and joined the DNSAP during his very first year of study. He also joined their paramilitary association, the *Volkssportverein*, comparable to the *Sturmabteilung* (SA) of the NSDAP. Sachs was also involved in the National Socialist German Student Union (NSDStB) at Charles University. In 1931, he became their university group leader [11, 17]. He was thus known as a National Socialist activist to the university administration, the professors, and the public. The Czech authorities watched the activities of the National Socialists with suspicion, arrested numerous members of the Volkssportverein in 1932, and initiated a trial [39].

In this context, Sachs and his comrades-in-arms sat in remand for 6 weeks, which later brought them the reputation of “martyrs” and “old fighters” of the NS movement. Sachs was finally released on his word of honor not to continue agitating against the state. The procedure was stopped [11, 17].

In addition to his manifold political activities and studies, Sachs worked as a volunteer and then as a demonstrator at the Institute of Pathology at the University of Prague from 1933. In 1935 he was employed there as a research assistant. In February 1936 he received his doctorate as Dr. med and in 1937 he also passed the examination for medical officer [6].

## The annexation of the Sudetenland

In the German Reich, Hitler consolidated his power after his appointment to Reich Chancellor in January 1933. One of his foreign policy objectives towards Czechoslovakia was the annexation of the Sudetenland. By signing the Munich Agreement in September 1938, Great Britain and France gave in to Hitler’s demands. The Sudetenland was occupied and incorporated by German troops until October 10, 1938. Sachs was a passionate supporter of the Sudetenland annexation and joined the Sudeten German Party in March 1938. He had also registered for the *Freiwilliger Schutzdienst *(Voluntary Protection Service), a paramilitary organization in which he held a leading position [6, 11, 17]. As already at the beginning of the 1930s, Sachs was thus not only a committed party member, but a true political activist who was ready to fight for the National Socialist agenda. Like the members of the Wehrmacht who had taken part in the occupation of the Sudetenland, Sachs received the Medal in Memory of October 1, 1938, colloquially known as the Sudetenland Medal [17], for his active role in this action.

Sachs continued to work as a research assistant at the Institute of Pathology at the German University of Prague. His research interests clearly showed how much he had become absorbed in Nazi ideology. In the winter semester of 1938/39, for example, he gave lectures on hereditary pathology and racial hygiene [6]. In December 1938, Sachs married Hildegard Krippner (born July 26, 1919), who was 5 years younger. She came from a Catholic family from the small village of Nedraschitz (*Nedražice*), about 25 km west of Pilsen (*Plzeň*). Her father worked as an authorized signatory in Prague. Hildegard shared her husband’s political convictions, belonged to the NS Women’s Association and the NSDAP (membership no. 7,077,758) [17].

After the “smashing of the rest of Czechia” and establishment of the Protectorate of Bohemia and Moravia in March 1939, German troops began to pursue and arrest German emigrants and communists as well as representatives of the Czech ruling elite. The German Charles University in Prague was soon assigned a new task: it was to serve as a research center for Southeast Europe and for “racial studies.” *Reichsdeutsche* (Germans from the Reich) were appointed to chairs and the subject “racial hygiene” was introduced [40]. Sachs swam on this wave and took over the department of *Volkspflege* (People’s cultivation) at the *Bund der Deutschen* (Federation of Germans), and gave public lectures on hereditary diseases [6]. Herwig Hamperl (1899–1976) took over the chair of pathology and the corresponding institute [33].

With the establishment of the protectorate, the Sudeten Germans received German citizenship, became subject to military surveillance, and were now able to join the Reich German Nazi organizations. Sachs had already applied for admission to the NSDAP on March 13, 1939, and became a party member retroactively as of November 1, 1938 (no. 6,863,383). In addition, he joined the SS of the NSDAP on June 20, 1939, receiving the membership number 337,622 [22].

Under Heinrich Himmler (1900–1945), the *Reichsführer-SS* and chief of the German police, the SS had grown into a powerful instrument of repression, which was also responsible for the concentration camp system. Sachs was given the rank of *SS-Oberscharführer*; he had served in the SS in *Sanitäts-Sturm XXXIX* in Prague (SS upper section Bohemia and Moravia). On his black SS uniform, he was allowed to sew the angle for *Alte Kämpfer* (old fighter). He left the Protestant Church and professed *Gottgläubigkeit* (belief in God), Himmler’s neo-pagan SS religion and one of the unofficial requirements for a career in the SS. In addition, he applied for the *SS-Zivilabzeichen* (civil badge; 169.398), which from then on decorated the lapel of his civilian suit in addition to the NSDAP party badge [13].

In a biography written for the SS in July 1939, Sachs presented himself as an early fighter for the National Socialists and highlighted his achievements and personal commitment [11]. Sachs was undoubtedly no stranger to Prague’s Nazi circles and received various honors. The NSDStB, for example, awarded him the medal of honor—in particular for his work as university group leader and honorary judge of the NSDStB [20].

## The Second World War

On September 1, 1939, the German Reich invaded Poland—the beginning of the Second World War. Sachs was promoted on this day to *SS-Untersturmführer* and thus to an SS officer of the *Allgemeine SS*. In the following weeks there were protests by Czech students at the University of Prague, which led to the closure of the Czech University in Prague in November 1939. Of the demonstrators, nine were executed and several hundred students and 55 professors and lecturers were deported to concentration camps [49]. It is not known whether Sachs was on site at this time, but the events cannot have remained hidden from him.

On January 15, 1940, Sachs was drafted into the Wehrmacht as a medical officer in the air force and received his basic training in the Luftwaffe Sanitäts-Ersatz Company in Baden near Vienna. From December 1940 to February 1941, he completed a course for reserve officers at the *Luftkriegsschule 1* (School for Aerial Warfare 1) in Dresden. Afterwards, he participated in the so-called Balkan campaign and the conquest of the island of Crete as head of the motorized field laboratory 2 and pathologist of an air force hospital at the medical service of air fleet 4 (*Unternehmen Merkur* [Operation Mercury]; May 20 to June 1, 1941). After the end of the fighting he was awarded the cuff “Crete” and the Iron Cross 2nd Class [17, 25]. Subsequently, Sachs was released from military service to teach at Charles University (indispensable/“uk”) and was able to continue working on his habilitation thesis in Prague [12, 14].

## German tyranny in the Protectorate of Bohemia and Moravia

In the so-called protectorate, the Germans had in the meantime established a reign of violence and terror. The appointment of Reinhard Heydrich (1904–1942) to deputy *Reichsprotektor* in Bohemia and Moravia in September 1941 led to the arrest of 6000 people and the execution of over 400 death sentences. Sachs obviously maintained good personal relations with the SS leadership there, especially with Heydrich’s deputy, the *Höherer SS- u. Polizeiführer Böhmen und Mähren* Karl Hermann Frank (1898–1946) [15]. On 30 January 1942, Sachs was promoted to *SS-Obersturmführer*. In the SS he was considered a “deserving *Volkstumskämpfer*” (fighter for German national identity), since he had already been actively engaged in this area for “German national identity” before the occupation of the Sudetenland. In the General SS, it was said that he looked after his men “with special zeal” in his function as leader of Sanitäts-Sturm XXXIX [16].

Heydrich was seriously wounded in an attack by Czech resistance fighters on May 27, 1942, and died on June 4 as a result of the attack. His body was examined at the Institute of Pathology. It can be assumed that Sachs assisted his superior and head of the institute, Professor Herwig Hamperl, who performed the autopsy [33]. In the following weeks a wave of arrests and terror swept over the protectorate, in which the Germans, among other things, destroyed the village of Lidice and shot the male inhabitants.

Sachs’ career at the university and in the SS developed rapidly. At the beginning of 1943 he habilitated in general pathology and pathological anatomy with his thesis “On autogenic pigments, especially lipofuscin and its distinction from melanin.” The work was rated “good” in every respect—both by the first reviewer, his boss Hamperl, and by Maximilian Watzka (1905–1981), director of the Histological Institute and SS comrade of Sachs [7, 8]. The *NS-Dozentenbund *strongly supported his appointment as a lecturer, since Sachs had “always been in the forefront of the fight for German nationality” [9]. This seemed to have been more important than his actual professional achievements, for Sachs had published only 11 papers in specialist journals between 1935 and 1943. There is no evidence of a detailed habilitation thesis, nor is it mentioned in his list of publications from the period after the war [60]. The appointment as lecturer by the Reich Minister for Science, Education, and National Education took place on June 11, 1943 [10].

In addition to his teaching activities, Sachs again served in the air force, namely at the Air Force Hospital in Prague. With the support of Karl Frank, Sachs was seconded to the SS hospital in Prague with effect from July 14, 1943, and finally, in the fall of 1943, he was transferred to the conduct of the business of the Chief Medical Officer SS and Police for the Höherer SS- und Polizeiführer Böhmen und Mähren Frank. In this relatively prominent position, Sachs also became acquainted with the Reichsarzt SS Ernst-Robert Grawitz. For the time being, he remained a member of the Luftwaffe. However, Frank and Grawitz had already supported a takeover in the Waffen-SS (armed SS) for some time [15]. In the early summer of 1944, Grawitz offered Sachs the position as chief pathologist at the Reichsarzt SS and Police as well as the simultaneous transfer to the Waffen-SS [20].

The exact tasks that were linked to this position are not documented. As a rule, membership in the staff was accompanied by advisory functions in the respective fields and the assumption of special tasks for the Reichsarzt SS [35], and at the same time meant a kind of distinction.

Furthermore, one can assume that the position was accompanied by the prospect of rapid promotions. After all, other members of the staff held general ranks in the SS, such as the chief dentist Hugo Blaschke [24], the SS medical supply quartermaster Carl Blumenreuter [48], and the chief SS surgeon Karl Gebhardt [35]. In comparison, Sachs had by far the lowest rank as a subordinate SS leader.

Sachs accepted the offer, agreed to the change, and was transferred to the Waffen-SS as SS-Obersturmführer with effect from May 3, 1944, i.e., he no longer belonged to the Luftwaffe and now wore the SS uniform. On June 21, 1944, he was promoted to *SS-Hauptsturmführer* of the Reserve of the Waffen-SS [19]. Himmler had also awarded him the SS skull ring [17]. With effect from May 1, 1944, Sachs was transferred from Prague to Berlin—to his new office, the *Stab Reichsarzt SS* [18].

## Secret chemical weapons program “N-Substance”

Within the framework of biological and chemical warfare, the Germans worked on the development and production of highly toxic nerve toxins such as tabun, sarin, and soman. From 1942, they also dealt with the military applications of the so-called *Normal-Stoff* (normal substance, N‑substance), which was chemically chlorine trifluoride or trichlorofluoride. This is a colorless gas that reacts with almost all organic and inorganic substances and corrodes or ignites them, including mucous membranes, eyes, and the respiratory tract of living beings, as well as metals, plastics, and concrete. Gas masks as well as bunkers, tanks, and airplanes would not have offered adequate protection against N‑substance as ammunition or as a chemical weapon during the Second World War. However, these actual effects had hardly been researched at that time and chlorine trifluoride was classified by the Wehrmacht as comparatively harmless [30, 43, 44, 47].

In March 1944, *SS-Gruppenführer* Karl Brandt (1904–1948), authorized representative for medical and health services, issued a Fuehrer’s order in which he stated the urgency of experiments with warfare agents. These included “lost experiments”—experiments in which about 50 Russian, Czech, and German prisoners had already been killed in the Natzweiler concentration camp in 1942/43 [1, 41]. Other tests concerned the compatibility of seawater, which the chief of the Air Force Medical Service in the Dachau concentration camp had proposed under the auspices of the air force. There were also tests on the threat to the water supply from warfare agents. In these tests, active acids intended for detoxification were used [2, 3, 38, 47, 61].

On July 7, 1944, Hitler ordered that Reichsführer-SS Heinrich Himmler should carry out further experiments with N‑substances as soon as possible [43, 44, 47]. Sachs had just taken over the position of chief pathologist at the Reichsarzt SS. Himmler assigned the head of the technical office of the Waffen-SS, *SS-Brigadeführer* Professor Dr. Otto Schwab (1889–1959), with further N‑Stoff research.

One area of research was the effect of N‑Stoff on humans [30, 43, 44, 47, 61]. Schwab asked Grawitz for the secondment of two doctors, “who should attend experiments with N‑substance as medical experts” [36]. Grawitz thereupon commanded SS-Hauptsturmführer Dr. Kurt Plötner (1905–1984), head of department at the *Institut für wehrwissenschaftliche Zweckforschung des SS-Ahnenerbes* (Institute for military Purpose Research of the SS-Ahnenerbe), as well as Sachs to test the effect of the N‑substance.

In September 1944, a first test was carried out to determine further research needs. No further information is available on this. The only evidence of this trial is a letter from the Reichsarzt SS in November 1944, in which concentration camp prisoners are requested for experiments with N‑substance [36].

As a result of the September experiment, the SS planned, with Himmler’s permission, to further investigate the effects of N‑substance in human experiments to test the suitability of N‑substance as a chemical weapon [30, 43, 44, 47, 61]. In November 1944, five concentration camp prisoners from Sachsenhausen were requested as test persons via the above-mentioned letter [36]. According to current sources it is only certain that there were further experiments with N‑substance, about which Plötner informed his superior. However, it cannot be clarified how these experiments were conducted [43, 44, 47]. It is very likely, however, that Plötner performed experiments on humans [44]. In a hearing in 1967, Plötner merely stated in his defense that no prisoners were harmed in the experiments with N‑substance, since the substance was allegedly harmless [47].

Since Sachs had no special expertise in the field of chemical warfare agents, he was presumably to collaborate in the experiments in his function as a pathologist, especially to test the effect of N‑substance on internal organs. Moreover, in his function as a member of the staff of the Reichsarzt SS, he was only obligated to the latter [35] and not to the staff of the SS-Ahnenerbe, which was responsible for the experiments. The Reichsarzt may have wanted him to be present as an independent reporter.

Whether Sachs was actually privy to the research with chemical substances or actively involved cannot be determined on the basis of the current file situation, since his name is not mentioned in the relevant documents. However, he certainly had more in-depth knowledge of the initial plans regarding the N‑substance tests and probably took part in the first test. Shortly after further experiments with N‑substance had taken place in late November 1944 or the first half of December 1944 [43, 44, 47], Sachs was transferred to the Western Front by the Reichsarzt SS [21]. It is possible that Sachs was involved in the experiments until he was given the new task (however, N‑substance experiments continued until early 1945 [44]). It is also possible, however, that he was no longer involved in the experiments and was transferred to the front due to the worsening war situation.

Grawitz applied to Himmler for further experiments with *Gelbkreuz* (mustard gas) and related warfare agents in February 1945, since he wanted to use prisoners here as well [4], but Himmler refused [5]. Ultimately, one can only speculate about the exact interests of the SS regarding the N‑substance. It is possible that the initiative came from individuals who wanted to make a name for themselves with Himmler [47], or that the SS Ahnenerbe or Himmler himself planned to gain access to German chemical weapons research via N‑Stoff [44].

## End of the war, internment, and denazification

In mid-December 1944, Sachs was transferred to the High Command of the 6th SS Panzer Army, the largest combat unit ever set up under an SS command. He was assigned to the army doctor in charge as consulting pathologist. The 6th SS Panzer Army led the attack focus of the Ardennes Offensive and was subsequently transferred to Hungary. However, the units had nothing left to oppose the Red Army and withdrew to Austria. Sachs described his activities in 1948: “I drove from one field hospital to another to see if the wounded were properly fed or if anything could be improved.” He claimed to have examined only corpses of people who had been medically treated for a long time, because everything else had fallen into the domain of the forensic doctor [50]. Together with the remains of the 6th SS Panzer Army, Sachs was taken prisoner of war by the United States on May 8, 1945, near Linz [25].

Among the Allies’ war aims were the punishment of Nazi crimes of violence and the denazification of the Germans. Unlike members of the Wehrmacht, who were considered regular prisoners of war, NS functionaries, potential war criminals, and SS members were subject to automatic arrest and were held in internment camps, to prevent an escape and to be able to investigate in detail.

Sachs was transferred to internment custody as SS-Hauptsturmführer on May 21. He passed through the American internment camps Haid (August 1945 to 1946), Gmunden (August 1946), Glasenbach (August 1946 to March 1947), Dachau (March to April 1947), and Kornwestheim (April to September 1947) [25, 53, 55].

In December 1946, the medical trial against leading Nazi doctors began. The human experiments in the concentration camps were also negotiated in this process [28]. Although the prosecution was in possession of the document already discussed in connection with the experiments on warfare agents, which mentioned Sachs’ name [36], the Allies apparently had no interest in questioning him. In any case, for various reasons, the subject of biological and chemical weapons was only marginally addressed in the Nuremberg trials [30]. It is possible that Sachs was not given too much importance, since his name was only mentioned in the documents at the beginning and since he was later demonstrably transferred to the front.

In the Kornwestheim camp, the public plaintiff of the camp’s verdict chamber started an investigation against Sachs. The decision was made to release Sachs from internment and to have the proceedings conducted by the local chamber of appeal. On September 16, 1947, Sachs was released to Bad Mergentheim in what is now Baden-Württemberg [53, 55]. His wife Hildegard had lived there since September 1945, having moved back from Prague to her hometown at the end of 1944, where her second child was born a week after the end of the war in May 1945. The Americans had already occupied the area by the end of April 1945. Probably with the withdrawal of US troops to the occupation zone established in Yalta and in view of a possible retaliation by the Soviets, she had fled with both daughters to Bad Mergentheim [54].

Sachs had to go through the denazification proceedings still pending before the Bad Mergentheim court. His defense strategy was simple: he claimed that he had only been commanded to the Waffen-SS, but had never really been a member of the Waffen-SS.

Because of the war he had only served 6 months in the SS anyway. In his experience, the SS was by no means a criminal organization. He stated that his membership in the NSDAP and various other Nazi organizations had to be seen in the light of the years of disenfranchisement of the German minority in the Czech Republic. Sachs was able to present a statement by his teacher Herwig Hamperl, who in the meantime had taken up a position at the Salzburg Regional Hospital. However, Hamperl was conspicuously reticent in his letter. He stated that Sachs had worked extremely conscientiously at the institute and had “never missed official hours or lectures for the purpose of working in the party or SS.” This was anything but a typical “clean bill of health” in the tone of a general apology. Hamperl rather used the letter to put his own institute in a good light. He wrote that he could not remember that Sachs had engaged in National Socialist propaganda at the institute. “This corresponded to my own attitude that a scientific institute was there for work and not for politicizing” [51]. Perhaps Hamperl did not want to risk too much at the time, since he was still working on his “clean slate” and posing as an apolitical scientist himself [33].

The public applicant requested, after inspection of the file, that Sachs be placed in the group of the main culprits (group I). However, the court was deceived by Sachs and held that he had lost everything as a displaced person. The SS leader and political activist had thus turned into a “victim of the world war.” Sachs was finally classified as a follower (group IV) and sanctioned with the payment of 50 RM [52]. With this denazification notice, he fulfilled the requirements to be reinstated into the civil service. Initially he worked as a general practitioner in Bad Mergentheim, before he became head of the bacteriological and serological examination department there for 3 months.

## Career in the Federal Republic of Germany—chair at the University of Münster

In 1948, Sachs was offered a position as a research assistant at the Institute for Forensic Medicine at the University of Münster via old networks from the Nazi era. The institute there had developed into a refuge for former SS members. Albert Ponsold (1900–1983), full professor of forensic medicine at the Reich University of Posen from 1941 to 1945, had taken over as director of the institute in 1948 and brought former SS men to Münster [26]. In September 1948, the Minister of Education and Cultural Affairs of the state of North Rhine-Westphalia had not yet considered a scheduled appointment of Sachs to be opportune in view of his SS past. However, in February 1949, she gave her approval after a meeting with Ponsold [58, 59]. It is a reality of contemporary history that after the founding of the Federal Republic of Germany, the social integration of even heavily incriminated Nazi perpetrators became one of the most important elements of Adenauer’s dealing with the past [29].

As a habilitated pathologist, Sachs now worked primarily on the implementation of forensic sections and the subsequent microscopic examination of the preserved body material. His scientific activity was mainly in the field of forensic pathology. On July 25, 1949, the Medical Faculty of the Westphalian Wilhelms University of Münster appointed Sachs as a private lecturer in forensic medicine. This was not a re-habilitation, but a renewed habilitation through recognition of his previous publications and activities. He gave his inaugural lecture on “Problems in the assessment of criminal abortion.” On April 23, 1951, he was appointed extraordinary professor. For a manual by Ponsold, Sachs contributed the chapters “Death by burning and scalding” and “Death by electricity” [45, 46]. The family had settled down well in Münster. After the birth of a son in Bad Mergentheim, two more children followed, so that the number of children had grown to five. Sachs was elected an extraordinary member of the International Academy for Forensic and Social Medicine in 1955 [60].

However, the good relationship between Sachs and Ponsold broke down in the mid-1950s due to disputes over the financial remuneration of private expert opinions. Ponsold was not only the director of the institute, but also ran a private practice, where expert opinions as well as blood group and blood alcohol analyses were carried out and invoiced. According to Ponsold, Sachs refused to give up part of the additional earnings thus gained to finance laboratory and personnel. On April 1, 1957, Sachs was made a civil servant as a lecturer and left the institute, and thus the institute’s budget, in a dispute. Since there was no chair available, a dietary lectureship was created for him, giving him an independent position as professor. However, his workplace was still located in the rooms of the Institute of Forensic Medicine. His monthly salary, with supplements, amounted to just under 1400 DM, making him one of the top earners in 1957.

When Sachs tried to obtain his own license as a blood group assessor, the conflict with Ponsold escalated. Ponsold saw his source of income in danger and refused to write Sachs a reference. Sachs responded by filing a disciplinary complaint. Furious, Ponsold wrote to the rector of the University of Münster that Sachs was only interested in his own profit, while he himself was using this money to finance four employees of the institute. He was to ensure that Sachs did not work in Ponsold’s domain. In a sharp tone, the director demanded that Ponsold write the job reference for Sachs, otherwise he would have to hand the matter over to the Minister of Education and Cultural Affairs. On the same day, Ponsold handed over a very good reference for Sachs. On July 31, 1958, Sachs was added to the list of blood group experts at the expert advisory service at the state level, and the announcement was made in the *Ministerialblatt* [56].

Thus, Sachs had a lucrative source of income in addition to his civil servant lectureship at the University of Münster. In the following years, Ponsold tried unsuccessfully to get rid of Sachs. For example, he suggested to the curator that Sachs be transferred to the RWTH Aachen University to teach engineering (Aachen did not yet have a medical faculty at that time). The curator then had to make it clear to Ponsold that such a decision fell within the competence of the Minister of Education and Cultural Affairs and that it would have to be preceded by an appeal from the university concerned [57].

In April 1967, Sachs was appointed scientific councilor and professor at the University of Münster. He took on teaching positions at the University of Bochum and the University Hospital in Essen as a paid part-time job. In 1968 Ponsold was finally retired, but subsequently tried by all means available to prevent Sachs from succeeding him. The position remained vacant for over 2 years, and all appointment negotiations failed. Finally, for purely factual reasons, the medical faculty decided to propose Sachs to the Ministry of Education as the first and only candidate (primo et unico loco). On December 15, 1970, Sachs was appointed full professor and took over as director of the Institute for Forensic Medicine at the Westphalian Wilhelms University of Münster.

The *Münstersche Zeitung* published a big report and introduced the scientist in more detail. There was not a word about his SS past; instead, it was mentioned that he had worked as a consulting pathologist in a Panzer Army and that he had fallen into American captivity (instead of internment). On March 31, 1980, Sachs was retired after reaching the age of 68 [56]. The *Westfälische Nachrichten* and the *Münstersche Zeitung* reported even later on the occasion of Sachs’ “round” birthdays [56]. On the occasion of his 80th birthday in 1992, there is also a tribute in the *Deutsches Ärzteblatt* [27], and an obituary can be found in *Rechtsmedizin* [23]. Sachs’ SS membership was still not mentioned with a word. Regarding the Nazi era and the war years, only his service as a medical officer in the Luftwaffe was noted. On September 11, 2000, Sachs died in Münster at the age of 88. The rector of the Westphalian Wilhelms University of Münster had an obituary placed in the *Westfälische Nachrichten*: with Sachs, the university had lost “a recognized researcher, a dedicated teacher and doctor, a highly esteemed person and colleague,” “who had rendered lasting services to science at the medical faculty.” The abridged biography showed considerable gaps for the period between 1933 and his return from “war captivity” [42].

## Conclusion

Hans Sachs belonged to the group of German university professors whose academic careers in the Third Reich received special protection and impetus due to their National Socialist convictions, their position in the system, and their activities. In 1944, Sachs joined the Waffen-SS and was involved in experiments with chemical warfare agents as chief pathologist in the staff of the Reichsarzt SS. Even if Sachs’ role in this context cannot be enlightened in detail, it could not have escaped his notice that in various experiments by the SS in the concentration camps, prisoners were murdered. Sachs must also have known the conditions in the concentration camps, since according to the Dachau prisoner doctor and pathologist Franz Blaha, he participated in an inspection of the Dachau camp in 1944 [37].

**Fig. 2:**
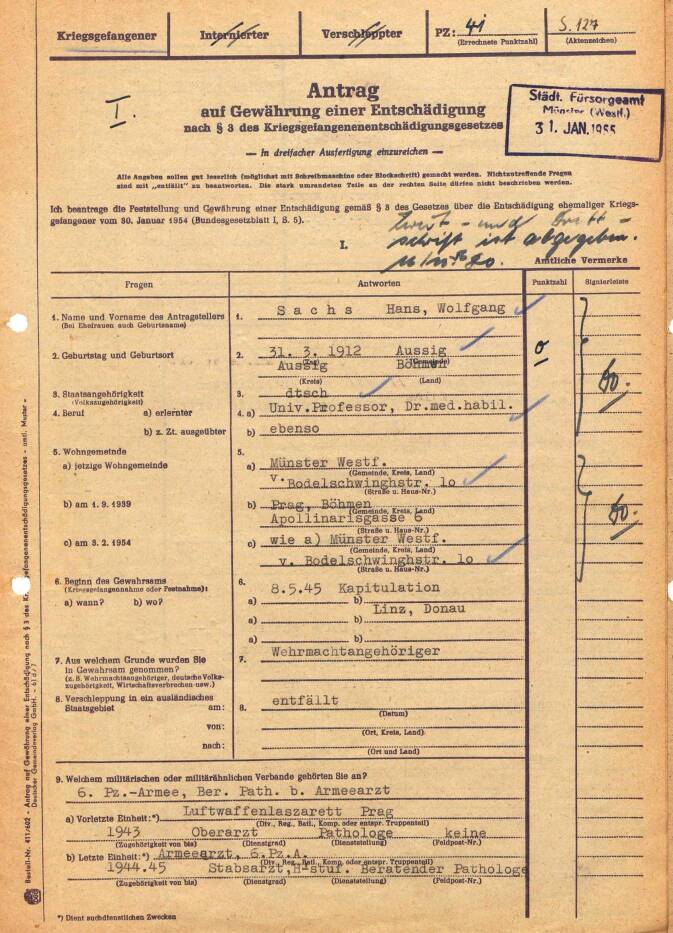
Compensation application of 31.01.1955 [55]. Sachs refers to himself as a member of the Wehrmacht, the term “SS” is deliberately not mentioned

Although he was heavily burdened politically as a National Socialist Volkstumskämpfer and SS leader, he managed to gloss over his biography after 1945 and ascribe an apolitical role to himself. He successfully portrayed his conversion to the Waffen-SS as an involuntary detachment; his activity as leading pathologist with the Reichsarzt SS was not discussed. Thus, he managed—like many other National Socialists [31], including pathologists [32, 33]—to pass through denazification without harm and to continue his professional career. Soon the term “service in the Waffen-SS” was changed slightly to “service in the Wehrmacht,” and “internment as a member of the SS” became a “normal” captivity of war (Fig. 2). In the young Federal Republic of Germany, many biographies of former SS members were “reinterpreted” in this way, thus disguising their own Nazi past [62]. Usually, there were no critical questions—on the contrary: well established “silence cartels” and intact networks from the Nazi era helped Sachs to pursue a career at the University of Münster.
